# Associations of maternal serum ferritin levels across gestation with gestational diabetes mellitus: A longitudinal cohort study

**DOI:** 10.1111/1753-0407.70027

**Published:** 2024-11-11

**Authors:** Huiqin Mo, Jingna Wen, Cuicui Qu, Xiaohua Liu

**Affiliations:** ^1^ Department of Obstetrics, Shanghai Key Laboratory of Maternal and Fetal Medicine, Obstetrics and Gynecology Hospital Tongji University School of Medicine Shanghai China; ^2^ Department of Obstetrics and Gynecology The Seventh People's Hospital of Shanghai University of Traditional Chinese Medicine Shanghai China

**Keywords:** gestational diabetes mellitus, iron supplementation, longitudinal changes, pregnant women, serum ferritin

## Abstract

**Background:**

The longitudinal changes in maternal serum ferritin (SF) levels across gestation, which indirectly reflect iron supplementation, have not been extensively investigated in relation to gestational diabetes mellitus (GDM).

**Methods:**

We conducted a retrospective cohort study at a tertiary maternal hospital in Shanghai. Women with SF concentration measurements at 8.0–13.6 weeks' gestation (GW), 29.0–31.6 GW, and an oral glucose tolerance test (OGTT) at 24–28 GW were included. We utilized logistic regression analysis to assess GDM association with maternal SF levels and longitudinal changes.

**Results:**

The study included 17 560 women, with 2160 (12.3%) participants diagnosed with GDM. Adjusted odds ratios (ORs) (95% confidence intervals [CIs]) for GDM across increasing quartiles of SF concentrations at 8.0–13.6 GW were 1.00 (reference), 1.139 (95% CI: 1.012–1.283), 1.093 (95% CI: 0.969–1.233), and 1.248 (95% CI: 1.111–1.403). Similarly, at 29.0–31.6 GW, increasing quartiles of SF concentrations were associated with higher adjusted ORs for GDM: 1.00 (reference), 1.165 (95% CI: 1.029–1.320), 1.335 (95% CI: 1.184–1.505), and 1.428 (95% CI: 1.268–1.607). Pregnant women with higher SF levels (upper 25th percentile) at 8.0–13.6 GW had a reduced GDM risk if their SF levels decreased to the lower 25th percentile at 29.0–31.6 GW. Conversely, the subgroup with higher SF levels (upper 25th percentile) at both time points had the highest incidence rate of GDM (15.3%, 1.235 [95% CI: 1.087–1.404]).

**Conclusions:**

Maternal SF levels independently and positively associated with GDM risk during early and late gestational stages. Considering the increased GDM risk, routine iron supplementation for iron‐replete women is questionable.

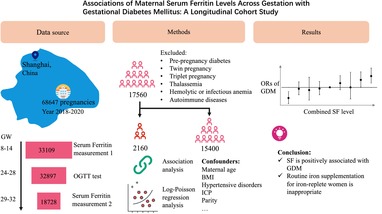

## Graphical abstract

**Highlights**

- This study was the first to explore the association between the longitudinal changes in maternal serum ferritin with GDM.
- We found pregnant women that initially elevated ferritin levels can reduce their risk of GDM by lowering their ferritin levels in later stages of pregnancy.
- Routine iron supplementation may not be suitable for women with sufficient iron levels.

## 1 INTRODUCTION

Gestational diabetes mellitus (GDM) is characterized by varying degrees of glucose intolerance during pregnancy and has become an enormous public health challenge globally, with prevalence ranging from 2% to 25% depending on geographic region and country.^1^, ^2^ GDM has been proved to be associated with significant adverse health consequences for both mother and offspring.

Prenatal iron supplementation is effective in preventing maternal iron deficiency and anemia during pregnancy. The World Health Organization (WHO) recommends daily iron supplementation of 30–60 mg for pregnant women due to the high prevalence of iron deficiency anemia (IDA) and advises measuring the serum ferritin (SF) concentration as the best screening test for iron stores in routine clinical examination.^3^, ^4^ Taking into account the increased iron demand that is exacerbated by the feto‐placental unit and the need to compensate for blood loss during delivery, more pregnancies focus on prenatal iron supplementation. However, it is often overlooked that this intervention may lead to excessive iron intake, which can catalyze the biochemical formation of reactive oxygen species that damage proteins, DNA, and lipids.^5^ Previous epidemiological evidence has raised critical concerns about GDM being accompanied by high SF level during pregnancy.^6^, ^7^, ^8^, ^9^, ^10^, ^11^, ^12^, ^13^, ^14^, ^15^ Even though GDM has been observed to be associated with higher SF levels, the impact of iron supplementation on pregnant women is an ongoing process. To our knowledge, only one prior case–control longitudinal study has examined iron status during pregnancy,^10^ and it concluded that ferritin levels were positively associated with GDM risk in both the first and second trimesters. However, this study did not investigate the longitudinal change in maternal plasma ferritin levels, which indirectly reflects iron supplementation during gestation. The routine iron supplementation may lead to adverse maternal and perinatal outcomes in some iron‐replete women, suggesting the necessity of using ferritin levels across pregnancy to guide iron supplementation.^16^ Therefore, we conducted this longitudinal cohort study to assess the association of maternal SF (across the entire range) at early and late gestational stages with GDM and to explore the association of longitudinal maternal SF levels changes with GDM.

## 2 METHODS

### 2.1 Data source and study participants

This retrospective cohort study was conducted among pregnant women registered at Shanghai First Maternity and Infant Hospital (SFMIH), Tongji University School of Medicine from May 30, 2018, to December 22, 2020. SFMIH is currently the largest obstetric care center both in Shanghai and in China, with 25 000–34 000 annual deliveries. Women who had SF concentrations measured at 8.0–13.6 weeks' gestation (GW), 29.0–31.6 GW, and oral glucose tolerance testing at 24–28 GW were included in the study. Women with pre‐pregnancy diabetes (type 1 or 2), multiple pregnancies, hemoglobinopathies, chronic infections, and those without information on GDM diagnosis or blood samples were excluded. This study was approved by the Ethics Review Board at SFMIH (KS21270).

Data were extracted from the hospital's electronic medical records systems, including information on prenatal fertility‐related characteristics, obstetric complications, labor and delivery summaries, and postpartum maternal and neonatal information. The prenatal information of the participants was obtained through a baseline questionnaire during the first prenatal visit (8.0–13.6 GW), which included blood pressure, maternal age, educational level, gravidity, parity, and medical history and medications. In subsequent prenatal visits, registered pregnant women will be screened for GDM between 24 and 28 GW, followed by serum iron level testing again between 29.0 and 31.6 GW. Pre‐pregnancy body mass index (BMI), categorized as <18.5, 18.5–24.0, 24.0–28.0, and >28 kg/m^2^, was calculated based on self‐reported pre‐pregnancy weight and height. Gestational weeks were estimated based on self‐reported last menstrual period and confirmed by first‐trimester ultrasound. GDM was diagnosed using the International Association of Diabetes and Pregnancy Study Groups (IADPSG) criteria. The one step 75 g, 2 h OGTT was performed at 24–28 weeks of gestation, GDM was diagnosed when any one or more glucose values met or exceeded the following cut‐offs: fasting ≥5.1 mmol/L, 1‐h ≥ 10.0 mmol/L, and 2‐h ≥ 8.5 mmol/L. SF levels were measured using a commercialized Maglumi ferritin immunoluminometric assay with a Beckman coulter Dxl 800 access machine at our institution. The reagents and calibrants used were from the same company, and the interassay coefficient of variation for this analysis was <5%.^17^, ^18^

### 2.2 Statistical analysis

The data were examined for missing and extreme values, as well as for logic checks. Descriptive statistics were presented as means and standard deviations for normally distributed variables, medians and ranges for skewed distributed variables, and numbers and percentages for categorical variables. Categorical variables were analyzed using a chi‐square test, while the independent sample *t*‐test was used to compare two groups with continuous variables that follow a normal distribution. For nonnormally distributed continuous data, the Mann–Whitney *U* test was used. Women had SF concentration measurements and blood sampling at 8.0–13.6 GW and subsequently repeat measurements at 29.0–31.6 GW. The quartiles of SF concentrations were defined based on the distribution of SF levels in two different trimesters. Women were stratified into four groups based on quartiles of SF concentrations (quartile 1 as the reference, quartile 4 as the highest) to examine whether SF levels at the two different trimesters were associated with GDM. To assess the association of longitudinal change in maternal SF levels with GDM, women were stratified into nine groups based on quartiles of SF concentrations at the two measurements: low (<25th percentile), intermediate (25th–75th percentile), and high (>75th percentile) in the two measurements, a 3 × 3 matrix, with the 2 × 2 group serving as the reference. The associations were assessed by calculating odds ratios (ORs) and their 95% confidence intervals (CIs) using log‐Poisson regression.^19^ Besides, the adjusted OR were obtained after adjusting potential confounding factors: age, pre‐pregnancy BMI, hypertensive disorders, intrahepatic cholestasis of pregnancy (ICP), parity, white cell count, and gestational weeks. Tests for trends were performed by fitting median values for each quartile of SF concentrations as a continuous variable. To model the association between continuous SF concentrations and ORs of GDM, 5‐knots restricted cubic spline regression was performed using the Stata (version 17.0; Stata Corp). Other analyses were performed using the SPSS software (version 26; IBM Corp, Chicago, IL). All the statistical tests were two‐tailed, and *p*‐values of <0.05 were considered statistically significant.

## 3 RESULTS

After excluding women with multiple pregnancies, pre‐pregnancy diabetes, hemoglobinopathies, and chronic infections, a total of 17 560 women met the inclusion criteria and were analyzed. Supplemental Figure S1 shows the flowchart of the study population. The characteristics of GDM and non‐GDM pregnant women are shown in Table 1. Women in the GDM group were older, heavier, and more likely to have complications including hypertensive disorders and ICP (Table 1). Hemoglobin (Hb) levels were higher in the GDM group at both measurement times, whereas the white blood cell (WBC) count in the GDM group were slightly higher at the first measurement and slightly lower at the second measurement. Furthermore, there were more cases of large for gestational age (LGA) and a decreased vaginal delivery rate in the GDM group. The incidence of premature rupture of membranes (PROM) was higher in the GDM group. However, the proportion of preterm birth and neonatal asphyxia (Apgar scores ≤7) showed no significant difference between the two groups.

**TABLE 1:** Baseline and obstetric characteristics of the study subjects.

Variable	GDM (*n* = 2160)	Non‐GDM (*n* = 15 400)	*p*
Maternal age, year^a^	32.6 ± 4.0	31.1 ± 3.7	<0.001
Prepregnancy BMI, kg/m^2^ ^a^	22.9 ± 3.4	21.6 ± 2.8	<0.001
<18.5 (*n*, %)	130 (6.0)	1643 (10.7)	‐
18.5–24.0 (*n*, %)	1359 (62.9)	11 202 (72.7)	
24.0–28.0 (*n*, %)	501 (23.2)	2114 (13.7)	‐
>28.0 (*n*, %)	170 (7.9)	441 (2.9)	‐
Parity ≥3, (*n*, %)	28 (1.3)	129 (0.8)	<0.001
Gestational week of delivery, week^a^	38.8 ± 1.2	39.2 ± 1.3	0.001
Birth weight, g			<0.001
<2500 (*n*, %)	73 (3.4)	422 (2.7)	–
2500–3500 (*n*, %)	1358 (62.9)	9983 (64.8)	–
3500–4000 (*n*, %)	589 (27.3)	4240 (27.5)	–
>4000 (*n*, %)	140 (6.5)	755 (4.9)	–
LGA (*n*, %)	158 (7.3)	653 (4.2)	<0.001
SGA (*n*, %)	75 (3.5)	465 (3.0)	0.0278
Hypertension diseases (*n*, %)	176 (8.2)	864 (5.6)	<0.001
ICP (*n*, %)	35 (1.6)	168 (1.1)	0.031
PROM (*n*, %)	312 (14.4)	2742 (17.8)	<0.001
Vaginal delivery (*n*, %)	1022 (47.3)	8536 (55.4)	<0.001
Preterm (*n*, %)	107 (5.0)	660 (4.3)	0.155
Apgar scores ≤ 7 (*n*, ‰)	19 (9.0)	124 (8.1)	0.716
Gestational week at first collection, week^b^	11.3 (8.0–13.6)	11.2 (8.0–13.6)	0.223
Gestational week at second collection, week^b^	30.6 (29.0–31.6)	31.1 (29.0–31.6)	<0.001
Hemoglobin at first collection, g/L^a^	128.8 ± 9.1	126.6 ± 8.9	<0.001
Hemoglobin at second collection, g/L^a^	118.2 ± 9.7	115.0 ± 9.3	<0.001
WBC count at first collection, 10^9^/L^a^	8.85 ± 2.1	8.26 ± 1.9	<0.001
WBC count at second collection, 10^9^/L^a^	8.83 ± 2.0	9.13 ± 2.0	<0.001

Abbreviations: BMI, body mass index; ICP, intrahepatic cholestasis of pregnancy; LGA, large for gestational age; PROM, premature rupture of membranes; SGA, small for gestational age; WBC, white blood cell.

^a^ Values are means ± SDs, and else are proportions for categorical variables.

^b^ Values are medians (minimum value‐maximum value).

**FIGURE 1:**
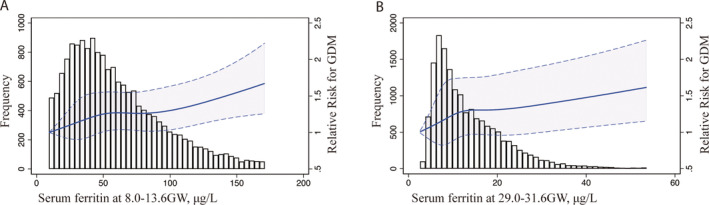
Relation between continuous serum ferritin and GDM risk. Splines represent adjusted ORs and 95% CIs of GDM in relation to serum ferritin concentrations (percentile 2.5–97.5; *n* = 16 682). The histogram represents frequency of participants according to SF concentrations. The solid line represents point estimates of ORs, and the dotted lines represent the 95%CI. ORs were calculated by the restricted cubic spline log‐Poisson regression model and were adjusted for maternal age (continuous), BMI (<18.5 kg/m^2^, 18.5–24.0 kg/m^2^, 24.0–28.0 kg/m^2^, >28.0 kg/m^2^) and ICP (yes or no) and hypertensive disorders (yes or no). BMI, body mass index; CI, confidence interval; GDM, gestational diabetes mellitus; ICP, intrahepatic cholestasis of pregnancy; OR, odds ratios; SF, serum ferritin.

The median (interquartile range [IQR]) concentration of ferritin in this study population was 51.7 μg/L (31.5–81.9 μg/L) at 8.0–13.6 weeks, which decreased to 11.3 μg/L (7.5–18.1 μg/L) at 29.0–31.6 GW. Table 2 shows the GDM risk associated with different SF categories among pregnant women. A significantly increased risk of GDM was observed among women with higher quartiles of SF during both trimesters, with those in the highest SF quartile (quartile 4, upper 25%) experiencing the highest risk of GDM. The unadjusted ORs of GDM increased with increasing quartiles of ferritin at 8.0–13.6 GW (p for trend <0.001) and at 29.0–31.6 GW (p for trend <0.001). The adjusted OR (95% CI) of GDM in the highest quartile of ferritin at 8.0–13.6 GW was 1.248 (1.111, 1.403) after adjusting for age, pre‐pregnancy BMI, hypertensive disorders, ICP, parity, gestational week, and WBC count at first testing. The adjusted OR (95% CI) of GDM in the highest quartile of ferritin was 1.428 (1.268, 1.607) at 29.0–31.6 GW. Figure 1 demonstrates the continuous association between GDM risk and the SF level at 8.0–13.6 GW (Figure 1A) and 29.0–31.6 GW (Figure 1B) using regression splines. The graph visually illustrates the increasing odds of GDM incidence with elevated SF levels during two gestational periods after adjusting for age, pre‐pregnancy BMI, hypertensive disorders, ICP, parity, gestational week, and WBC count at each testing.

**TABLE 2:** Associations of SF level with gestational diabetes risk.

Variables	GDM (*n*, %)	Unadjusted^a^ OR (95% CI)	Adjusted^b^ OR (95% CI)
Quartiles of plasma ferritin at 8.0–13.6 GW			
Quartile 1 (<31.5 μg/L)	480 (10.9)	1 (ref)	1 (ref)
Quartile 2 (31.5–51.7 μg/L)	548 (12.5)	1.144 (1.020, 1.283)	1.139 (1.012, 1.283)
Quartile 3 (51.7–81.9 μg/L)	521 (11.9)	1.094 (0.974, 1.226)	1.093 (0.969, 1.233)
Quartile 4 (>81.9 μg/L)	611 (14.0)	1.280 (1.145, 1.432)	1.248 (1.111, 1.403)
P for trend^c^	–	<0.001	0.001
Quartiles of plasma ferritin at 29.0–31.6 GW			
Quartile 1 (<7.5 μg/L)	458 (10.1)	1 (ref)	1 (ref)
Quartile 2 (7.5–11.3 μg/L)	500 (11.6)	1.148 (1.019, 1.294)	1.165 (1.029, 1.320)
Quartile 3 (11.3–18.1 μg/L)	575 (13.1)	1.296 (1.155, 1.455)	1.335 (1.184, 1.505)
Quartile 4 (>18.1 μg/L)	627 (14.4)	1.422 (1.270, 1.592)	1.428 (1.268, 1.607)
P for trend^c^	–	<0.001	<0.001

Abbreviations: BMI, body mass index; GDM, gestational diabetes mellitus; ICP, intrahepatic cholestasis of pregnancy; SF, serum ferritin.

^a^ Odds ratios (ORs) and their 95% confidence intervals (CIs) were calculated using log‐Poisson regression.

^b^ Log‐Poisson regression adjusted for maternal age (continuous), BMI (<18.5 kg/m^2^, 18.5–24.0 kg/m^2^, 24.0–28.0 kg/m^2^, >28.0 kg/m^2^) and ICP (yes or no) and hypertensive disorders (yes or no), and gestational week (continuous) and white blood cell count (continuous) at the collection time.

^c^ *P* for trends were performed by fitting median values for each quartile of SF concentrations as a continuous variable.

Table 3 shows the association of longitudinal changes in SF concentrations during two different trimesters with the incidence of GDM. Women with quartiles 2 and 3 of ferritin concentrations during both trimesters were combined as the reference group, known as the 2 × 2 group (comprising pregnant women with SF levels between 31.5–81.9 μg/L at 8.0–13.6 GW and then between 7.5 and 18.1 μg/L at 29.0–31.6 GW). Notably, a clear discontinuity in GDM risk was observed in forest plot, with the subgroup of women with quartile 1 both at 8.0–13.6 GW and 29.0–31.6 GW having the lowest incidence of GDM (9.0%, 0.777 [0.662–0.914]), while the incidence increased to 12.6% if the quartile 1 at first measurement increased to quartile 4 at second measurement. Pregnant women with higher SF at 8.0–13.6 GW had similar GDM risks, compared to the referent, if their SF level decreased to the lower quartile (quartile 1). Meanwhile, the subgroup of women in quartile 4 at both 8.0–13.6 GW and 29.0–31.6 GW demonstrated the highest GDM incidence (15.3%, 1.235 [1.087–1.404]).^20^

**TABLE 3:** The combined effect of serum ferritin concentrations during two different trimesters on GDM.

Groups^a^ of SF at 8.0–13.6 GW	Groups^a^ of SF at 29.0–31.6 GW	GDM (*n*, %)		Adjusted^b^ OR (95% CI)	*p*
1 (<31.5 μg/L)	1 (<7.5 μg/L)	171 (9.0)	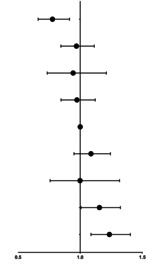	0.777 (0.662, 0.914)	0.002
	2 (7.5–18.1 μg/L)	251 (12.3)		0.970 (0.845, 1.113)	0.666
	3 (>18.1 μg/L)	58 (12.6)		0.943 (0.735, 1.211)	0.647
2 (31.5–81.9 μg/L)	1 (<7.5 μg/L)	240 (11.0)		0.974 (0.846, 1.121)	0.712
	2 (7.5–18.1 μg/L)	566 (12.1)		1 (ref)	
	3 (>18.1 μg/L)	263 (13.9)		1.087 (0.950, 1.244)	0.226
3 (>81.9 μg/L)	1 (<7.5 μg/L)	47 (10.9)		0.999 (0.758, 1.317)	0.996
	2 (7.5–18.1 μg/L)	258 (13.2)		1.155 (1.007, 1.324)	0.039
	3 (>18.1 μg/L)	306 (15.3)		1.235 (1.087, 1.404)	0.001

Abbreviations: BMI, body mass index; GDM, gestational diabetes mellitus; ICP, intrahepatic cholestasis of pregnancy; OR, odds ratios; SF, serum ferritin.

^a^ Pregnant women were stratified into nine groups based on quartiles of SF concentrations at two measurements (low, <25 percentile; intermediate, 25–75 percentile; and high, >75 percentile in two measurements; 3 × 3; with the 2 × 2 group as the reference).

^b^ Log‐Poisson regression adjusted for maternal age(continuous), BMI (<18.5, 18.5–24.0 kg/m^2^, 24.0–28.0 kg/m^2^, >28.0 kg/m^2^) and ICP (yes or no) and hypertensive disorders (yes or no).

## 4 DISCUSSION

The large longitudinal cohort study yielded three key findings: (1) Maternal ferritin levels exhibit a marked decline from early to late gestational stages. (2) Maternal ferritin levels are positively associated with GDM risk during both early and late gestational stages, independently. (3) The risk of GDM among women with high ferritin levels in early pregnancy will return to normal if their ferritin levels decrease to a lower level in late pregnancy. However, if their ferritin levels remain high, the risk of GDM remains the highest.

Ferritin is a reliable parameter for clinically determining baseline iron stores, differentiating IDA from other hypochromic anemias and diagnosing iron overload.^21^, ^22^ According to our routine clinical practice, SF and hemoglobin (Hb) levels were measured to monitor iron stores during the first prenatal visit (8.0–13.6 GW) and again in the third trimester (29.0–31.6 GW) following OGTT screening, which provided us the chance to investigate longitudinal ferritin levels changes during pregnancy. Our longitudinal data indicate a significant decrease in ferritin and hemoglobin levels from early to late gestational stages, which is consistent with the majority of previous research.^23^, ^24^ The physiological decrease in SF levels during pregnancy can be attributed to several main factors, including increased iron demand by the feto‐placental growth, elevated red blood cell mass, and the plasma volume expansion.^25^, ^26^

Some studies investigated the relationship between SF levels and the risk of GDM during at either early or late gestational stages and observed a positive association. To investigate this association in a Chinese population, we utilized a large data‐set derived from electronic medical records comprising 17 560 pregnancies, and observed maternal ferritin levels are positively associated with GDM risk during both early and late gestational stages, independently. Our longitudinal cohort in maternal SF addresses the limitations of previous studies with smaller sample sizes and one‐point measurement.^6^, ^7^, ^8^, ^9^, ^10^, ^11^, ^12^, ^13^, ^14^, ^15^ Although the underlying mechanism has not been clarified, several researchers have sought to shed light on the relationship between SF concentration and GDM. Fernandez‐Real et al. revealed a significant correlation between SF with insulin resistance.^27^, ^28^ Auvinen's study indicated that lower Hb levels in mice and humans are associated with improved glucose tolerance, healthier key metabolic measures, and blood pressure. These benefits are mediated by the tissue oxygenation status, involving the activation of the hypoxia inducible factor pathway.^29^

For pregnant women, routine use of iron supplementation has been shown to reduce the prevalence of maternal anemia at delivery.^30^ A proposed guideline suggests that women of reproductive age with SF levels <15 μg/L may be suffering from iron storage deficiency and potential IDA, necessitating iron supplementation, while those with SF levels ≥70 μg /L are not in need of supplementation.^31^ To prevent maternal IDA, the practice of universal iron supplementation during pregnancy is common in the United States and many other countries.^32^ In China, obstetricians may advise iron supplementation for pregnant women with SF concentrations <30 μg/L. In our study, only 23.1% of participants presented ferritin concentrations <30 μg/L in the first trimester, while this proportion increased to 93.8% in the third trimester. when it comes to diagnosing IDA, approximately 25.4% of pregnant women had Hb levels <110 g/L at 29.0–31.6 GW. It is obvious that SF concentrations <30 μg/L cannot be used to guide iron supplementation during late trimester. The decrease trend of ferritin levels across gestation implicates that different SF levels should be used to evaluate the iron store at different trimester, rather than the single criteria.

The therapeutic effect of iron supplementation during pregnancy, which depends on body iron stores, food, and the capacity of iron absorption, remains uncertain. Since assessing the exact iron intake of the body is difficult, we used increased quartiles of SF levels, along with elevated Hb concentrations, as indirect indicators of the efficacy of iron supplementation. This allowed us to examine whether iron supplementation in iron‐replete women could harm glucose tolerance. We found that the risk of GDM among women with high ferritin levels in early pregnancy returned to normal if their ferritin levels decreased to a lower level in late pregnancy. However, if their ferritin levels remained high, the risk of GDM remained the highest, demonstrating the excessive iron can affect glucose metabolism. Excessive iron initiates an overabundance of reactive oxygen species, which is considered a common denominator for placental pathologies of GDM.^5^ Serum hepcidin, a central regulator of iron metabolism, was also observed to increase in GDM, along with increased ferritin and iron levels.^33^ Several studies have found that high Hb levels increase blood viscosity and impair placental perfusion and that iron supplementation increases the risk of deficiencies in other minerals.^34^, ^35^ Xu Zhang et al. conducted a prospective cohort study to assess the impact of SF concentrations in early pregnancy combined with supplemental iron in midpregnancy on GDM risk. The study found that elevated SF levels and supplemental iron intake of ≥60 mg/day were independently associated with an increased risk of GDM.^36^ More prospective cohort studies about the baseline iron stores and dosage of iron intake across pregnancy are needed.^37^, ^38^, ^39^ Our results suggest that women with lower plasma ferritin concentrations (quartiles 1 and 2) at 8.0–13.6 GW will benefit from iron supplementation and are less likely to develop GDM. For women at quartiles 3 and 4 of SF in the early gestation, iron supplement may be harmful with respect to the increased risk of GDM.

Our study has some limitations. First, while SF is commonly used to assess iron stores, its diagnostic utility is limited since it also increases during acute inflammation. To address this issue, we excluded women with anemia of inflammation, characterized by SF levels >100 μg/L coexisting with Hb levels <110 g/L. We adjusted for major confounders identified in previous studies, such as maternal age, pre‐pregnancy BMI, hypertensive disorders, ICP, gestational week, and WBC count at the collection time. Due to the limitation of our retrospective nature, other potential confounding factors such as lipid^20^, ^40^ and uric acid^41^ cannot be included. The future prospective studies incorporating a broader range of parameters will provide a more comprehensive analysis. Second, due to the retrospective nature of present study, we could not obtain exact information regarding iron supplementation such as dosage and duration. Therefore, we could only rely on longitudinal changes in maternal SF levels across gestation, which provided an indirect assessment of iron supplementation efficacy. After being diagnosed with GDM, pregnant women were suggested to follow nutritional advice to manage their dietary patterns, such as reducing meat intake and adjusting carbohydrate consumption. These changes may potentially affect iron intake and lower SF levels at the second measurement point, which actually makes our finding of the association stronger.

In conclusion, our study demonstrates a positive association between SF levels and GDM risk among pregnant women, highlighting the importance of monitoring iron status during pregnancy. Women with lower plasma ferritin concentrations at 8.0–13.6 GW are advised to take iron supplementation, which does not increase the likelihood of developing GDM. Our findings also suggest that a decrease in SF levels across gestation may have a protective effect against GDM in some women. Considering the increased risk of GDM, routine iron supplementation for these iron‐replete women is questionable.

## AUTHOR CONTRIBUTIONS

Xiaohua Liu and Cuicui Qu designed research; Jingna Wen and Xiaohua Liu collected data; Huiqin Mo and Jingna Wen analyzed data, and Cuicui Qu, Huiqin Mo, and Xiaohua Liu wrote the paper. Xiaohua Liu had primary responsibility for final content. All authors read and approved the final manuscript.

## CONFLICT OF INTEREST STATEMENT

The authors declare no conflicts of interest.

## Supporting information


**Figure S1.** Flowchart of the study population.

## Data Availability

Data described in the manuscript, code book, and analytic code will be made available upon request pending approval by author Xiaohua Liu (annaabcd114@hotmail.com) and a signed data access agreement.
