# Supplementary figures and images for: Associations of maternal serum ferritin levels across gestation with gestational diabetes mellitus: A longitudinal cohort study
Source: J Diabetes. 2024 Nov 11;16(11):e70027. doi: 10.1111/1753-0407.70027 (PMC11551590; doi:10.1111/1753-0407.70027)

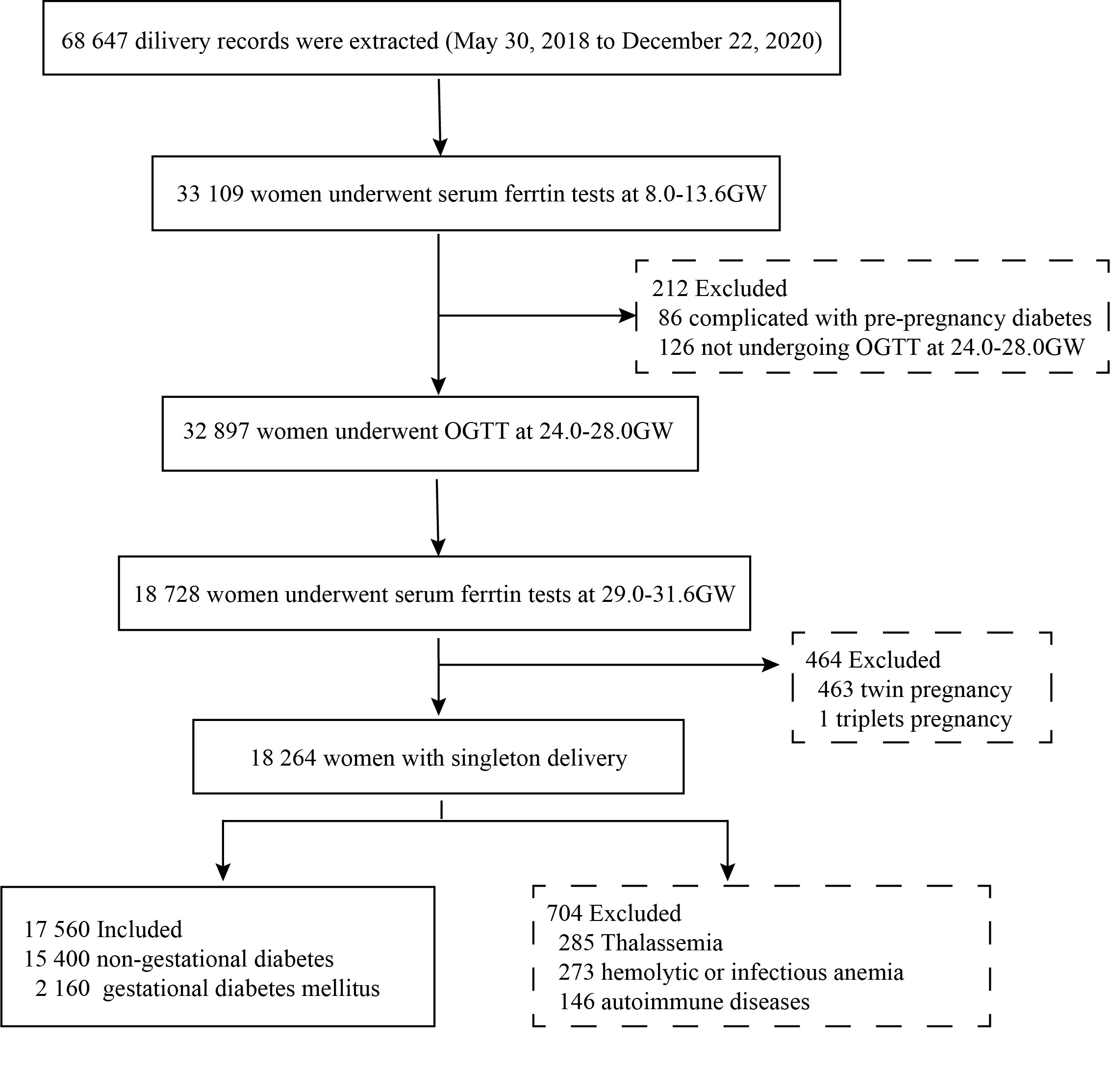

Supplement: Supplementary file 1 — Figure S1. Flowchart of the study population. [file JDB-16-e70027-s001.jpg]
